# NR4A2 protects cardiomyocytes against myocardial infarction injury by promoting autophagy

**DOI:** 10.1038/s41420-017-0011-8

**Published:** 2018-02-15

**Authors:** Honghong Liu, Pingping Liu, Xingxing Shi, Deling Yin, Jing Zhao

**Affiliations:** 1 Shandong Provincial Key Laboratory of Animal Cells and Developmental Biology, School of Life Science, Shandong University, Jinan, 250100 China; 2 Yantai Affiliated Hospital of Binzhou Medical University, Yantai 264000, China; 3 School of Pharmaceutical Sciences, Central South University, Changsha, 410078 China; 4 Department of Internal Medicine, College of Medicine, East Tennessee State University, Johnson City, TN 37614 USA

## Abstract

Myocardial infarction (MI), characterized by ischemia-induced cardiomyocyte apoptosis, is the leading cause of mortality worldwide. NR4A2, a member of the NR4A orphan nucleus receptor family, is upregulated in mouse hearts with MI injury. Furthermore, NR4A2 knockdown aggravates heart injury as evidenced by enlarged hearts and increased apoptosis. To elucidate the underlying mechanisms of NR4A2-regulated apoptosis, we used H9c2 cardiomyocytes deprived of serum and neonatal rat cardiomyocytes (NRCMs) exposed to hypoxia to mimic ischemic conditions in vivo. As NR4A2 knockdown aggravates cardiomyocyte apoptosis, while NR4A2 overexpression ameliorates it, NR4A2 upregulation was considered an adaptive response to ischemia-induced cardiomyocyte apoptosis. By detecting changes in LC3 and using autophagy detection tools including Bafilomycin A1, 3MA and rapamycin, we found that NR4A2 knockdown promoted apoptosis through blocking autophagic flux. This apoptotic response was phenocopied by downregulation of NR4A2 after autophagic flux was impaired by Bafilomycin A1. Further study showed that NR4A2 binds to p53 directly and decreases its levels when it inhibits apoptosis; thus, p53/Bax is the downstream effector of NR4A2-mediated apoptosis, as previously reported. Changes in p53/Bax that were regulated by NR4A2 were also detected in injured hearts with NR4A2 knockdown. In addition, miR-212-3p is the upstream regulator of NR4A2, and it could downregulate the expression of NR4A2, as well as p53/Bax. The mechanism underlying the role of NR4A2 in apoptosis and autophagy was elucidated, and NR4A2 may be a therapeutic drug target for heart failure.

## Introduction

Myocardial infarction (MI), characterized by ischemia-induced cardiomyocyte death, is one of the leading causes of death worldwide^1^. Cardiomyocytes are terminally differentiated cells, and cardiomyocyte death irreversibly damages the structure and function of the heart and leads to myocardial fibrosis, heart failure and other adverse cardiac events^2,3^. There are two kinds of cell death in MI injury: necrosis and apoptosis^4^. As apoptosis can be regulated by signaling pathways, a better understanding of the mechanisms modulating cardiomyocyte apoptosis is critical for identifying effective treatments for patients with ischemic heart diseases.

Autophagy, a well-conserved intracellular lysosomal degradation process, is particularly important to the homeostasis of terminally differentiated cells^5^. It is reported that autophagy occurs in ischemic cardiomyocytes to provide essential energy for cell survival by clearing disordered organelles or aging proteins^4^. A moderate amount of autophagy is beneficial to cell survival, while excessive autophagy may lead to cell death^6,7^. Given the complex crosstalk between apoptosis and autophagy, the key factors involved in apoptosis and autophagy still need more research for the development of potent drug targets^8,9^.

Nuclear receptor subfamily 4, group A, member 2 (NR4A2), also known as Nurr1, is a member of the NR4A orphan nuclear receptor family with NR4A1 and NR4A3^10^. The NR4A family belongs to the immediate early response transcription factors, which can quickly respond to ischemic stroke^11^. NR4A2 participates in stress-induced apoptosis of various cancer cells with different functions, indicating that it has a cell-specific role in apoptosis^12^. The role of NR4A2 in cardiomyocyte apoptosis has remained elusive. Furthermore, a recent report showed that NR4A2 increased in association with cell autophagy in neuronal survival^13^, but its association with autophagy remains unknown. Here, we aimed to clarify the function of NR4A2 in cardiomyocyte apoptosis and autophagy in order to determine whether it is a potential therapeutic target for ischemic heart diseases.

Given that NR4A2 may play an important role in ischemic cardiomyocytes, understanding its regulation is important. MicroRNAs (miRNAs) are a group of non-coding single-stranded RNA molecules that can bind to target messenger RNAs (mRNAs), resulting in a decrease in their translation^14^. In this study, the preliminary miRNA microarray results associated with predictive analysis by TargetScan suggested that miR-212-3p was the potential miRNA targeting NR4A2. MiR-212-3p possesses highly conserved sequences among vertebrates, and its expression is high in both the brain and heart, suggesting that it has an important role in the heart. Most research on miR-212-3p has focused on its roles in the development of tumors and neurons, but its function in heart has been less reported^15,16^. It has been reported that miR-212-3p promoted angiogenesis to attenuate ischemic heart disease by inhibiting the expression of Rasa1/Spred1, thus upregulating Ras/mitogen-activated protein kinase^17^. Currently, the function of miR-212-3p in ischemia-induced cardiomyocyte apoptosis is unknown.

Here, we studied the role of NR4A2 in ischemia-induced cardiomyocyte apoptosis and autophagy and reported a miR-212-3p/NR4A2/p53/Bax pathway in autophagy-dependent apoptosis in ischemia-stimulated cardiomyocytes.

## Results

### NR4A2 knockdown aggravated MI-induced heart injury

**Fig. 1:**
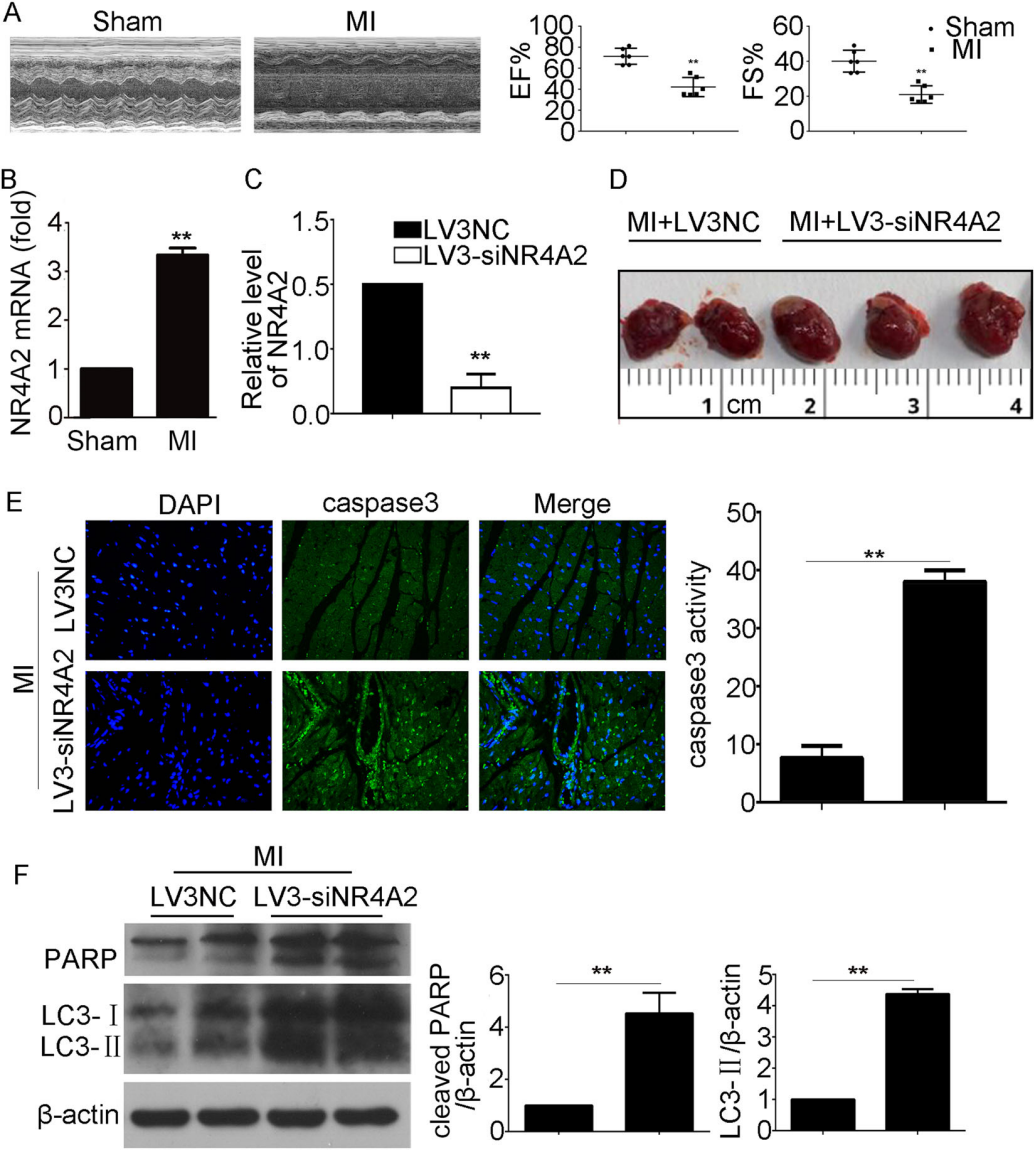
NR4A2 knockdown aggravated MI-induced heart injury. **a** The heart function was analyzed by echocardiography and the changes of EF and FS were revealed. **b** NR4A2 expression in sham and MI mice was detected by qPCR. Mice with MI injury were injected with lv3-siNR4A2 or controls for 7 days, and then the hearts were harvested. **c** The knockdown effect of lv3-siNR4A2 was detected by qPCR. **d** Gross morphology of the heart. **e** Caspase3 was detected by immunofluorescence. **f** PARP and LC3 levels were observed by western blotting. Data are expressed as the mean + S.D. **P < 0.01; *n* = 6

Seven days of permanent coronary ligation caused severe cardiac dysfunction, as we previously reported (Fig. 1a)^18^. NR4A2 expression increased in mouse hearts with MI injury as detected by real-time quantitative PCR (qPCR; Fig. 1b). We injected the interfering lentivirus of NR4A2 (lv3-siNR4A2) or negative control into hearts with MI. The qPCR results verified the knockdown effect of lv3-siNR4A2 in vivo (Fig. 1c). After 7 days of permanent coronary ligation, compared with control mice, mice injected with lv3-siNR4A2 exhibited enlarged hearts (Fig. 1d), and lv3-siNR4A2 increased the expression of the apoptosis markers poly(ADP-ribose) polymerase (PARP) and caspase3, as well as the autophagy marker LC3 (Fig. 1e, f), suggesting that NR4A2 knockdown aggravates MI-induced heart injury.

### Upregulated expression of NR4A2 in ischemic cardiomyocytes

**Fig. 2:**
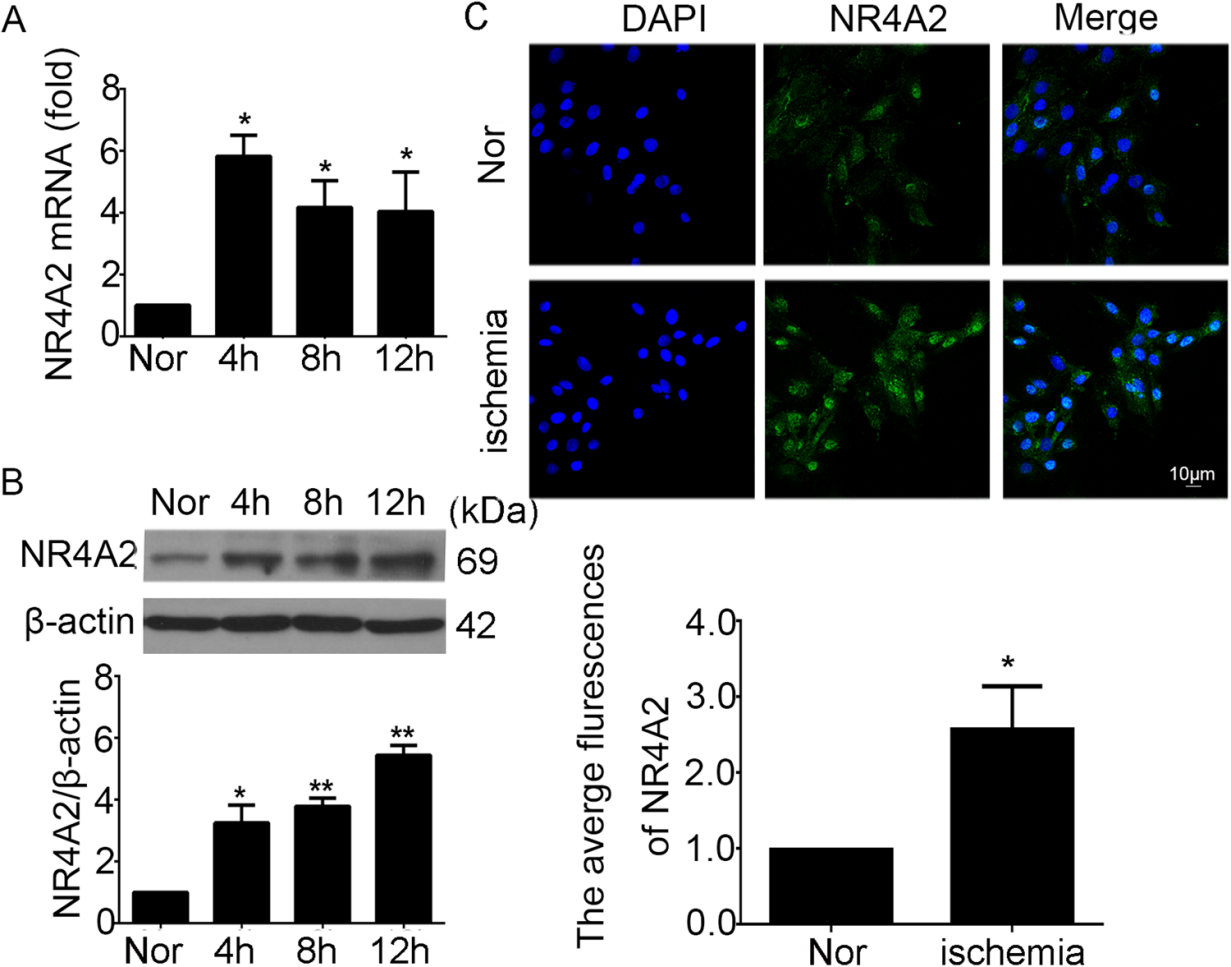
Expression of NR4A2 increased in cardiomyocytes exposed to ischemic conditions. **a**, **b** H9c2 cells were deprived of serum for 4 h, 8 h and 12 h, and the expression of NR4A2 was analyzed by **a** qPCR and **b** western blot. **c** H9c2 cells were cultured exposed to ischemic conditions (deprived of serum for 12 h), and the expression of NR4A2 was observed by immunofluorescence. Nor normal. Data are expressed as the mean + S.D. *P < 0.05; **P < 0.01; *n* = 3

H9c2 cardiomyocytes deprived of serum were used to mimic ischemic conditions^19^. To investigate the possible involvement of NR4A2 in cardiomyocyte apoptosis, we analyzed the changes in NR4A2 in H9c2 cardiomyocytes exposed to ischemia. When ischemia lasted for more than 4 h, NR4A2 increased as shown by qPCR, western blot and immunofluorescence (Fig. 2a–c).

### NR4A2 protected cardiomyocytes from apoptosis

**Fig. 3:**
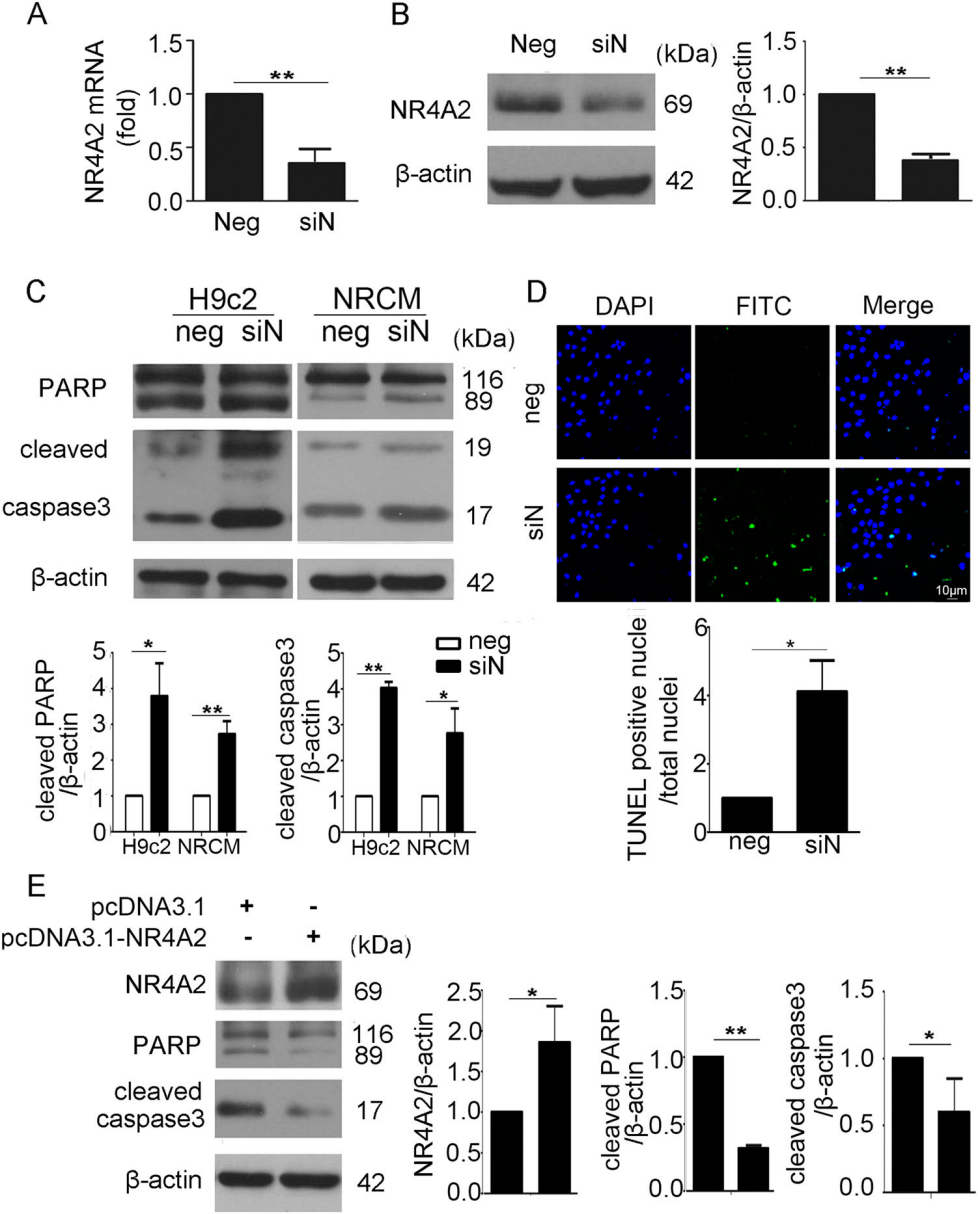
NR4A2 protects against ischemia-induced apoptosis in H9c2 cells and NRCMs. **a**, **b** H9c2 cells and NRCMs were transfected with NR4A2 siRNA; the interference effect of siRNA was detected by **a** qPCR and **b** western blot. **c** After knockdown of NR4A2, the expression of PARP and cleaved caspase3 in H9c2 cells and NRCMs exposed to ischemic conditions was observed by western blot. **d** Apoptosis-positive cells were detected by TUNEL staining to show the ratio of apoptosis in H9c2 cells exposed to ischemia and treated with or without NR4A2 siRNA. **e** H9c2 cells were transfected with pcDNA3.1-NR4A2 overexpression plasmid and then exposed to ischemic conditions (deprived of FBS for 12 h). Protein levels of NR4A2, PARP and caspase3 were detected by western blot. Neg negative control, siN siNR4A2. Data are expressed as the mean + S.D. *P < 0.05; **P < 0.01; *n* = 3

To evaluate the function of NR4A2 in ischemia-induced cardiomyocyte apoptosis, we used NR4A2 small interfering RNA (siRNA) to knockdown its expression. The qPCR and western blot analyses were used to verify the knockdown effect of NR4A2 siRNA in cardiomyocytes (Fig. 3a, b). Then, H9c2 cardiomyocytes and neonatal rat cardiomyocytes (NRCMs) exposed to ischemia were both used to detect the effects of NR4A2 knockdown on ischemia-induced cardiomyocyte apoptosis. The levels of cleaved PARP and caspase3 in H9c2 cardiomyocytes and NRCMs with NR4A2 knockdown were both higher than those in controls (Fig. 3c), and terminal deoxynucleotidyl transferase dUTP nick end labeling (TUNEL) staining showed more Fluorescein isothiocyanate (FITC)-positive cells in the NR4A2 knockdown group than in the control group (Fig. 3d), suggesting that NR4A2 knockdown promoted cardiomyocyte apoptosis. In addition, we used overexpression to verify the functions of NR4A2. As shown, compared with NR4A2 knockdown, NR4A2 overexpression had the opposite effect (Fig. 3e); thus, NR4A2 protects cells from ischemia-induced cardiomyocyte apoptosis.

### NR4A2 enhanced autophagic flux in cardiomyocytes

**Fig. 4:**
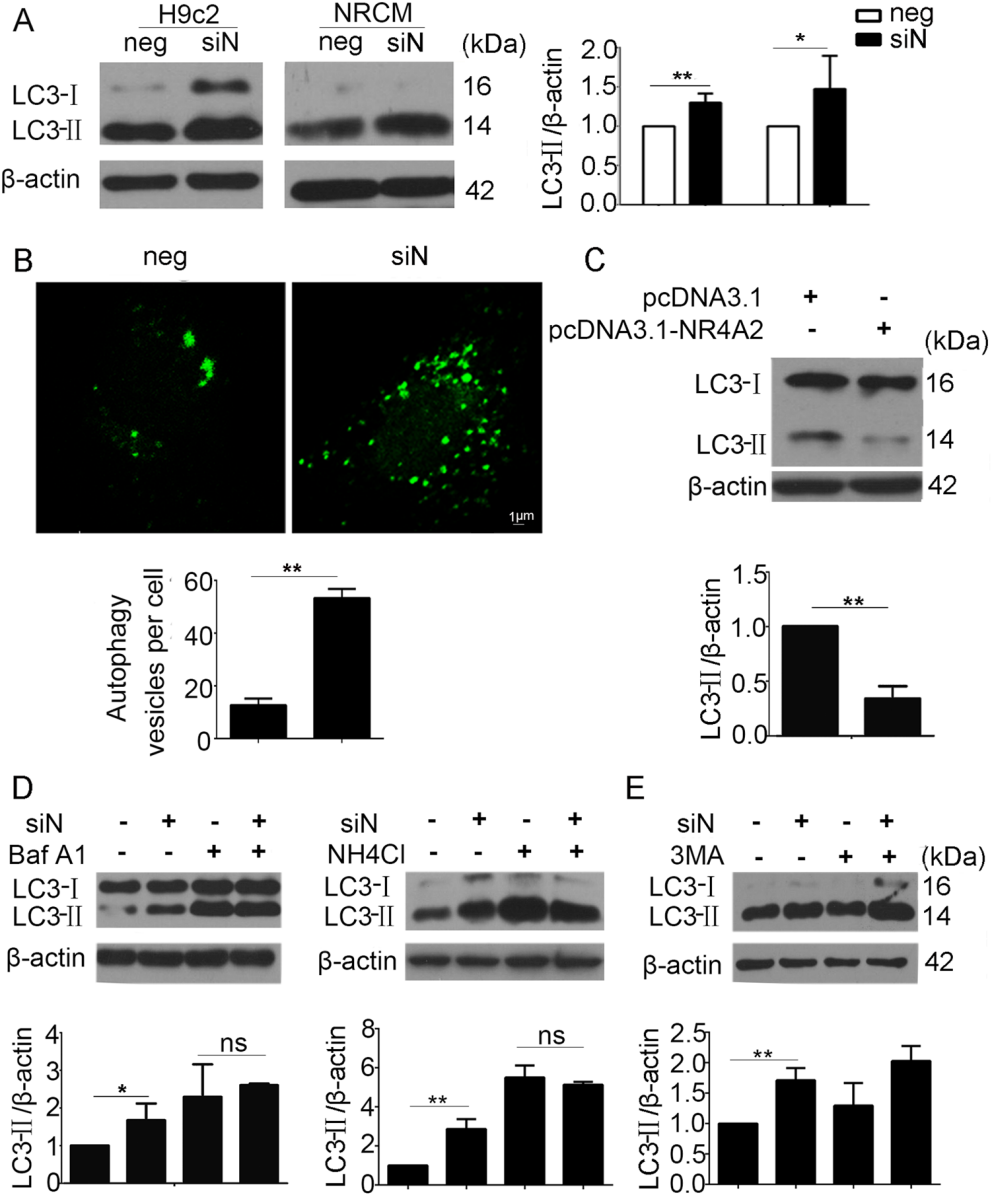
NR4A2 promotes autophagic flux of cardiomyocytes exposed to ischemic conditions. **a** H9c2 and NRCMs were transfected with NR4A2 siRNA followed by ischemia treatment as methods. Changes in LC3 levels were analyzed by western blot. **b** H9c2 cells exposed to ischemia were co-transfected with pEGFP-LC3 plasmid and NR4A2 siRNA, and then LC3 patches were revealed by immunofluorescence. **c** H9c2 cells were transfected with pcDNA3.1-NR4A2 overexpression plasmid for 48 h followed by serum deprivation for another 12 h, and then changes in LC3 levels were analyzed by western blot. **d**, **e** H9c2 cells were transfected with NR4A2 siRNA for 36 h, followed by treatment with autophagy blockers (Baf A1, NH_4_Cl and 3MA) in the ischemic condition for another 12 h. Then, LC3 levels were detected by western blotting. Baf A1 Bafilomycin A1, Rapa rapamycin. Data are expressed as the mean + S.D. *P < 0.05; **P < 0.01; *n* = 3

Autophagy occurs in hearts with MI injury, and it is now considered a therapeutic target for ischemic heart disease^20^. Recent studies reported that NR4A2 might be involved in autophagy^21^. In this study, NR4A2 knockdown increased ischemia-induced LC3-II upregulation in H9c2 cardiomyocytes and NRCMs and LC3 patches in H9c2 cells (Fig. 4a, b), suggesting that NR4A2 knockdown changed cardiomyocyte autophagy. Decreases in LC3-II levels after NR4A2 overexpression further confirmed that NR4A2 regulates autophagy (Fig. 4c).

To further confirm the change in LC3-II caused by autophagic initiation promotion or autophagic flux blocking, we used the autophagy inhibitors Bafilomycin A1 (Baf A1), NH_4_Cl and 3-methyladenine (3MA). Western blot results showed that when autophagic flux blockers Baf A1 or NH_4_Cl were added, there was significant augmentation of LC3-II in negative controls, and NR4A2 knockdown could not increase LC3-II further (Fig. 4d). In addition, 3MA could not decrease LC3-II levels in the NR4A2 knockdown group (Fig. 4e), suggesting that NR4A2 knockdown blocked autophagic flux but did not affect autophagy initiation.

### NR4A2 knockdown promoted cardiomyocyte apoptosis by inhibiting autophagic flux

**Fig. 5:**
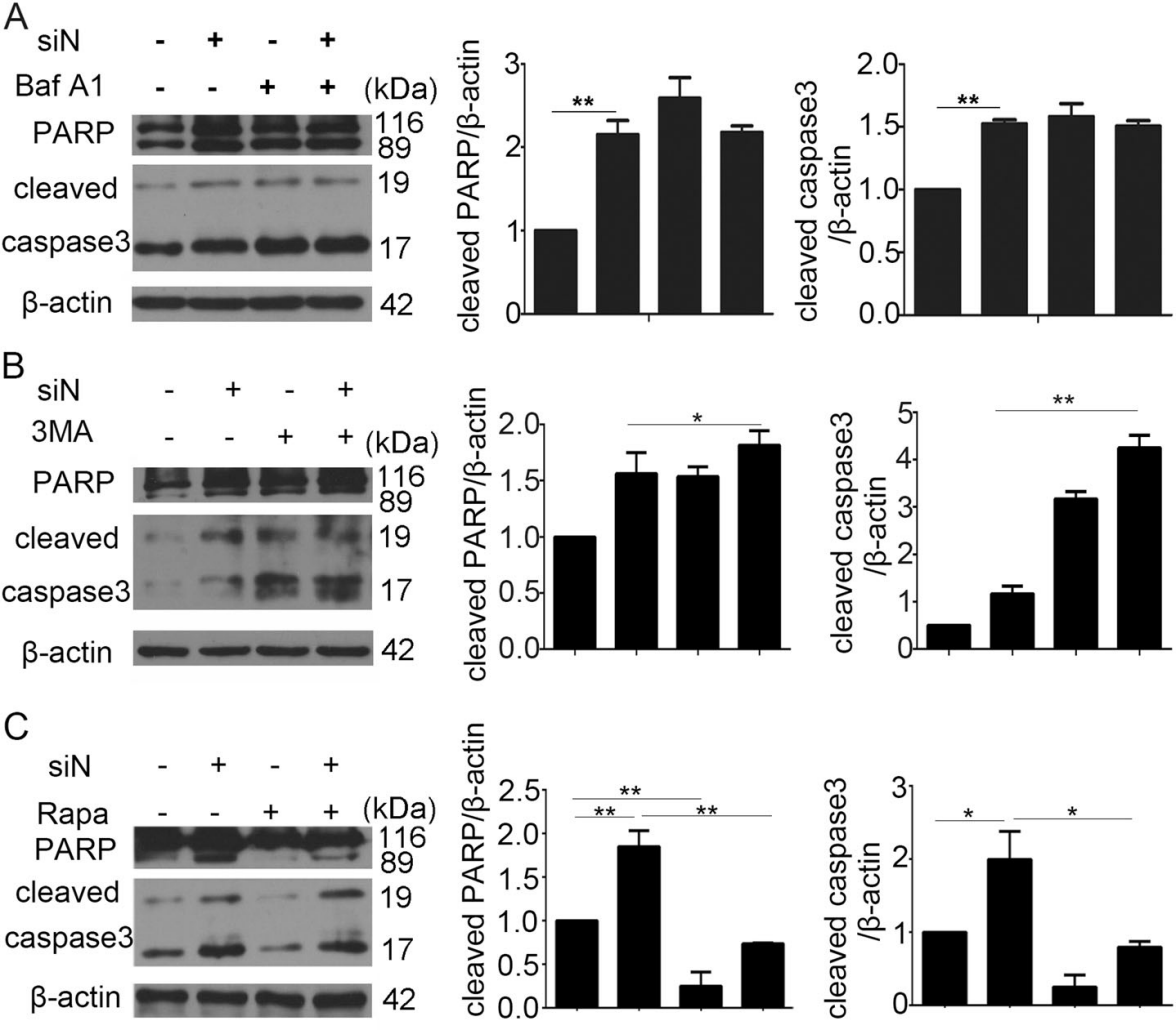
NR4A2 knockdown increased the apoptosis of H9c2 through blocking autophagic flux. H9c2 cells were transfected with NR4A2 siRNA for 36 h followed by serum deprivation. **a–c** Bafilomycin A1, rapamycin and 3MA were added separately to H9c2 cells deprived of serum. After 12 h, the PARP and cleaved caspase3 levels were detected by western blot. Data are expressed as the mean + S.D. *P < 0.05; **P < 0.01; *n* = 3

To elucidate the function of NR4A2-mediated autophagy on myocardial apoptosis, we used autophagy tools including Baf A1, 3MA and rapamycin. After the autophagy inhibitor 3MA was added, the levels of cleaved PARP and caspase3 induced by NR4A2 knockdown further increased (Fig. 5a), while the autophagy promoter rapamycin significantly decreased the levels of cleaved PARP and caspase3 (Fig. 5b), suggesting that NR4A2 knockdown promoted apoptosis via autophagy inhibition. Because impeding the autophagic flux was the key to inhibiting autophagy, the autophagic flux blocker Baf A1 was added to cells with NR4A2 knockdown. As a result, the levels of cleaved PARP and caspase3 were not changed in Baf A1-treated NR4A2 knockdown cells compared with the levels in cells with NR4A2 knockdown alone, suggesting that the two groups used the same mechanism to induce apoptosis; that is, NR4A2 knockdown promoted apoptosis by blocking autophagic flux (Fig. 5c).

### NR4A2 inhibited apoptosis through interaction with p53

**Fig. 6:**
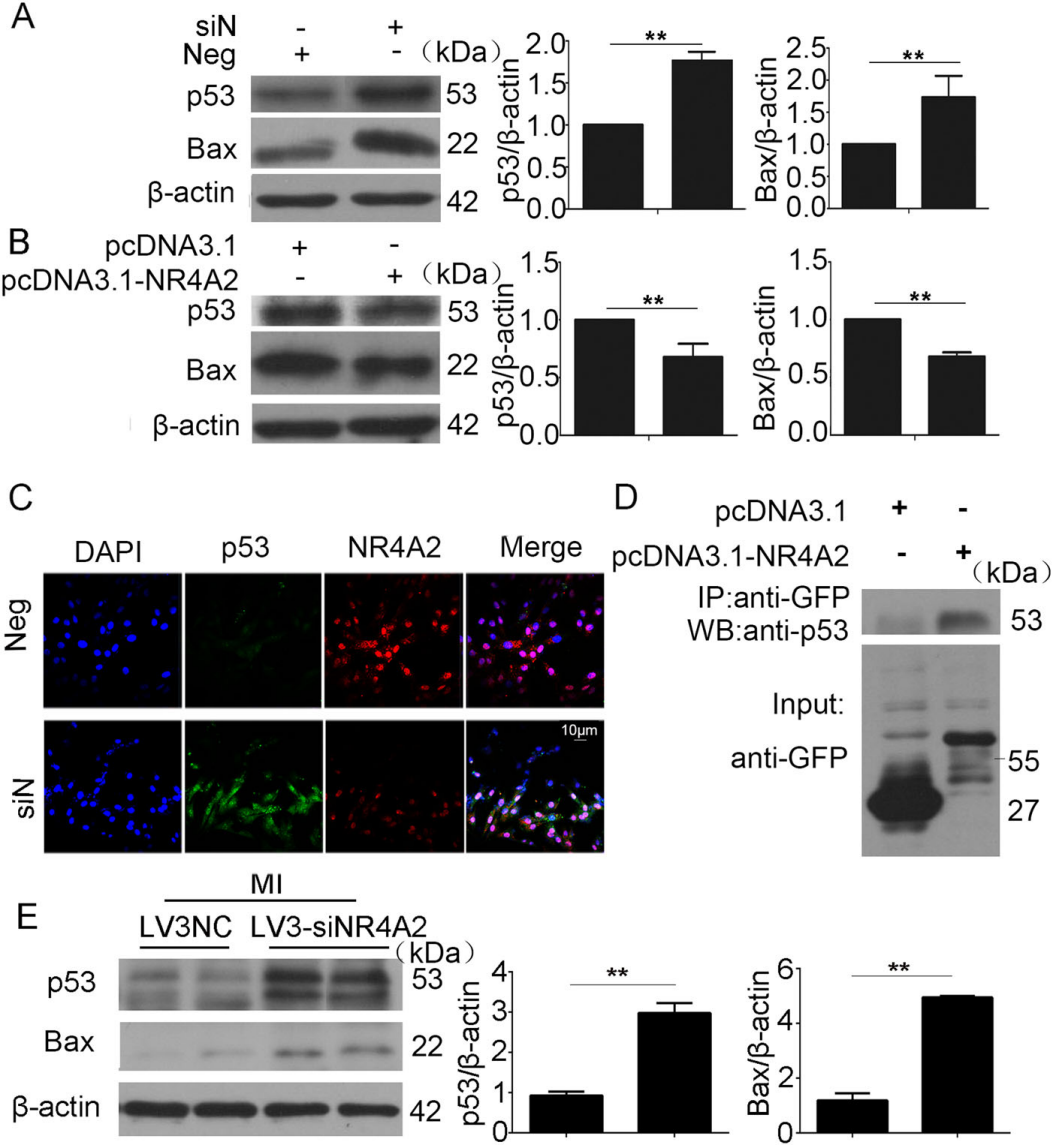
NR4A2 decreased p53 levels and bound to p53. **a**, **c** H9c2 cells were transfected with NR4A2 siRNA for 36 h followed by serum deprivation for another 12 h; then, p53 and Bax levels were analyzed by western blot (**a**). The possible colocalization of p53 and NR4A2 was detected by immunofluorescence (**c**). **b**, **d** H9c2 cells were transfected with pcDNA3.1-NR4A2 overexpression plasmid or vector plasmid followed by serum deprivation; then, p53 and Bax levels were analyzed by western blot (**b**), and the interaction of p53 and NR4A2 was detected by co-IP assay (**d**). **e** After MI for 7 days, the expression levels of p53 and Bax in the mouse heart with or without NR4A2 knockdown were detected by western blot. Data are expressed as the mean + S.D. *P < 0.05; **P < 0.01; *n* = 3

P53 might be the downstream factor of NR4A2 that inhibits Bax expression and induces apoptosis^22^. We used NR4A2 siRNA and an overexpression plasmid to detect the relationship between NR4A2 and p53. Western blot results showed that NR4A2 knockdown increased p53 and Bax levels, while NR4A2 overexpression decreased these levels (Fig. 6a, b). Co-immunoprecipitation (Co-IP) results revealed that p53 could bind to NR4A2 (Fig. 6d). We also used immunofluorescence to verify the colocalization of NR4A2 and p53 (Fig. 6c). The data revealed that p53 was the direct downstream target of NR4A2. To ascertain whether NR4A2 could regulate p53 and Bax in vivo, p53 and Bax levels in MI mouse hearts with NR4A2 knockdown were observed by western blot (Fig. 6e), and the same result was observed as in vitro.

### NR4A2 was the target of miR-212-3p

**Fig. 7:**
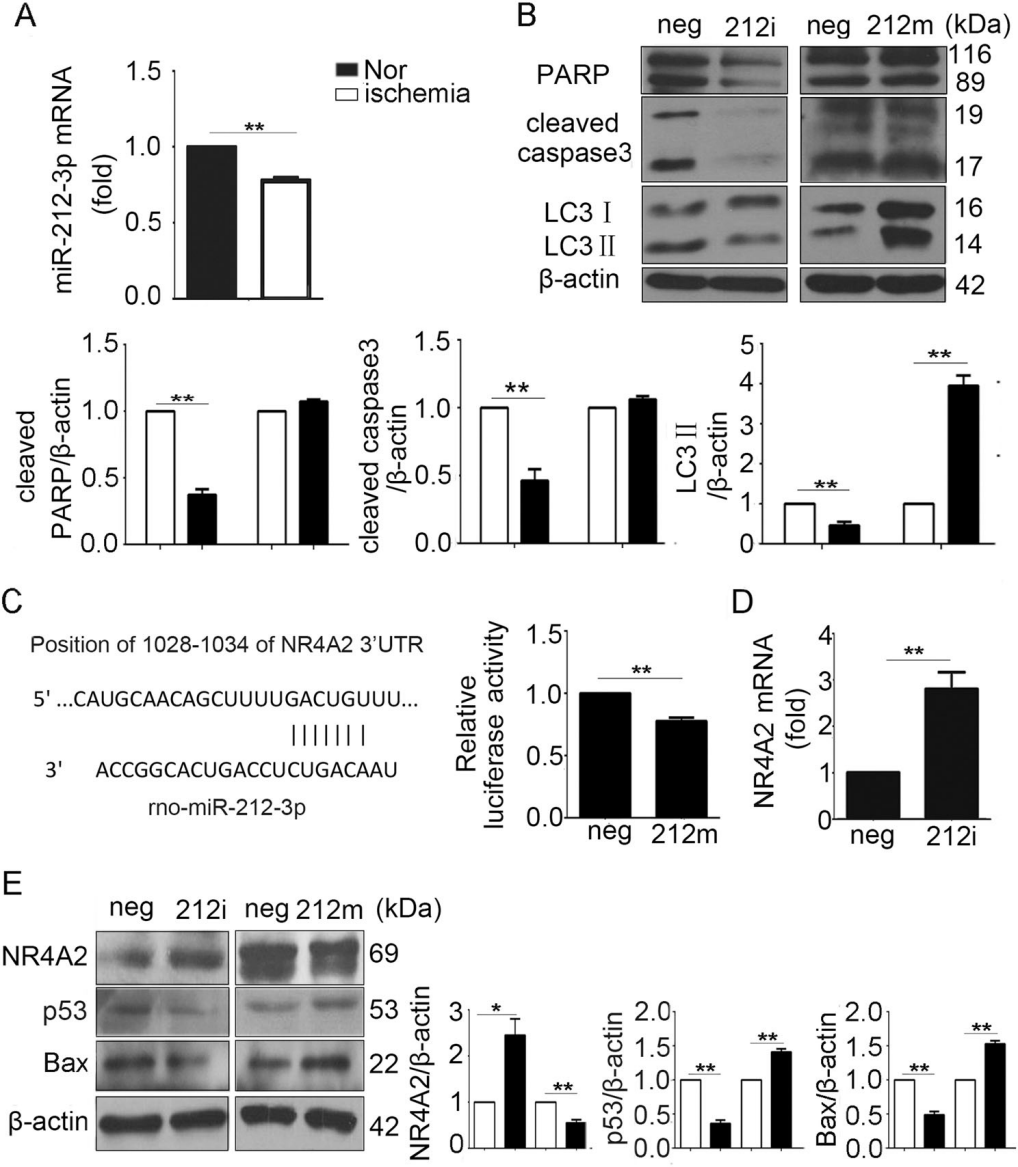
NR4A2 was the target of miR-212-3p in cardiomyocytes. **a** H9c2 cells were cultured in ischemic conditions (deprived of serum for 12 h). Expression of miR-212-3p was analyzed by qPCR. **b** H9c2 cells were transfected with inhibitor or mimic of miR-212-3p followed by serum deprivation for 12 h. Changes in PARP, caspase3 and LC3 were analyzed by western blot. **c** Website-predicted target of miR-212-3p and luciferase reporter plasmid containing the native NR4A2 3’UTR was co-transfected with miR-212-3p mimic in HEK293 cells for 24 h followed by a dual-luciferase activity assay. **d** H9c2 cells were transfected with miR-212-3p inhibitor in ischemic conditions, and NR4A2 levels were detected by qPCR. **e** H9c2 cells were transfected with miR-212-3p inhibitor or mimic in ischemic conditions, and then, NR4A2, p53 and Bax were detected by western blot. Data are expressed as the mean + S.D. *P < 0.05; **P < 0.01; *n* = 3

NR4A2 plays an important role in autophagy and apoptosis, but the mechanism regulating NR4A2 is still unknown. Bioinformatics tools (http://www.targetscan.org) predicted that miR-212-3p was a putative miRNA targeting NR4A2 based on target sequences at the NR4A2 3′ untranslated region (UTR). First, miR-212-3p expression and function was detected in cardiomyocytes exposed to ischemia. The qPCR showed that miR-212-3p decreased in ischemic conditions (Fig. 7a). An inhibitor of miR-212-3p suppressed the cleavage of PARP and caspase3, and thus it inhibited cardiomyocyte apoptosis as NR4A2 did, whereas a miR-212-3p mimic did not affect the above-mentioned apoptosis markers, perhaps because apoptosis was saturated (Fig. 7b).

**Fig. 8:**
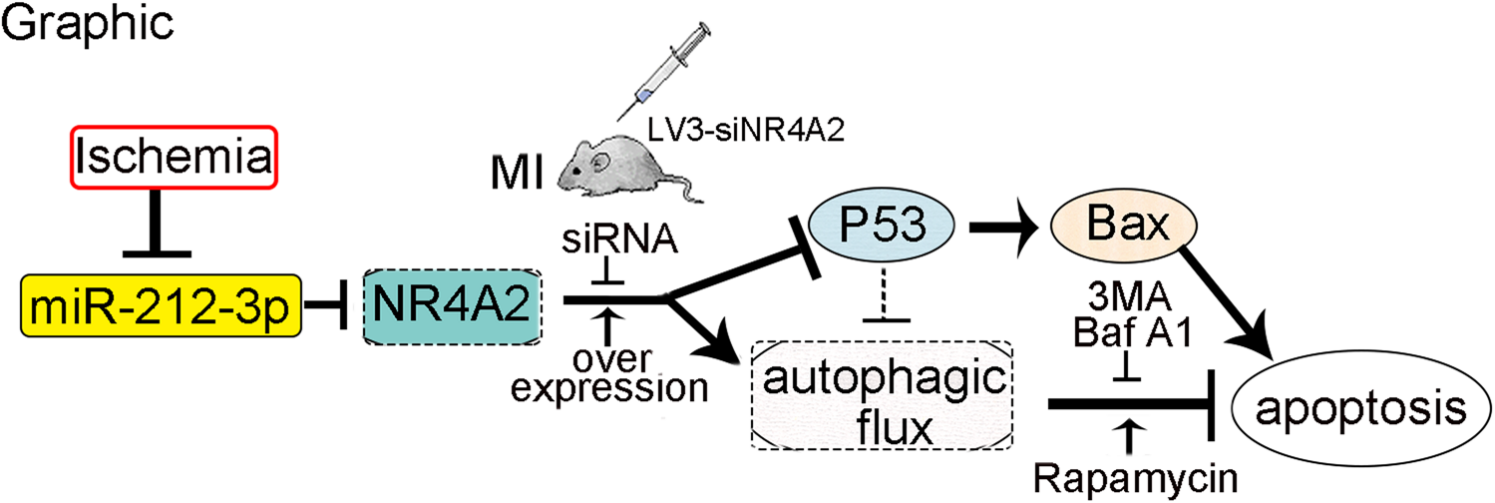
The graphic image shows that ischemia inhibited the expression of miR-212-3p, thus increasing its target NR4A2 levels. NR4A2 protected cardiomyocytes from apoptosis through inhibiting p53/Bax and enhancing autophagic flux

To verify the regulation of NR4A2 by miR-212-3p, we cloned the wild-type 3′UTR sequence of NR4A2 into a luciferase reporter vector (Fig. 7c), and then we performed a luciferase reporter assay to determine whether miR-212-3p could directly regulate the expression of NR4A2 in H9c2 cardiomyocytes (Fig. 8). The results showed that the luciferase activity linked to the wild-type 3′UTR of NR4A2 was repressed in H9c2 cells transfected with the miR-212-3p mimic compared with that in control cells (Fig. 7c). The qPCR and western blot results showed that the miR-212-3p mimic inhibited NR4A2 –expression (Fig. 7d, e), as well as the expression of p53 and Bax. All the data verified that miR-212-3p is the upstream regulator of NR4A2 (Fig. 7c–e).

## Discussion

Ischemic heart disease is one of the leading causes of mortality worldwide^1^. Cardiomyocyte apoptosis stimulated by a deficiency of oxygen and nutrients is the main mechanism leading to heart failure^23,24^; thus, understanding how to inhibit cardiomyocyte apoptosis is important for designing effective heart failure therapies^2,4^. In our study, NR4A2 was upregulated in murine cardiomyocytes exposed to ischemia. Further study revealed that NR4A2 knockdown could aggravate, while overexpression of NR4A2 could inhibit apoptosis stimulated by ischemia. Our data suggested that induction of NR4A2 is an adaptive response to resist cardiomyocyte apoptosis caused by ischemia. Furthermore, NR4A2 knockdown in MI mice caused apoptosis and heart myocardial hypertrophy. It has been reported that NR4A2 plays important roles in cancer, obesity, diabetes and neurological disease; our results reported a new role for NR4A2 in ischemic heart diseases^25–28^.

During ischemia, depleted serum triggered cardiomyocyte autophagic flux to supply essential energy^29,30^. In this case, autophagy is considered an adaptive reaction with cardioprotective effects. We found that NR4A2 induced the above-mentioned protective autophagy. First, NR4A2 was also triggered by serum depletion, which is the same as ischemia-induced autophagy. Second, NR4A2 regulated ischemia-induced autophagic flux. In the current study, cardiomyocyte apoptosis caused by ischemia was attenuated by NR4A2-enhanced autophagy, suggesting that NR4A2 might be used as a potential therapeutic target for ischemic heart diseases.

The molecular mechanisms regulating NR4A2-induced apoptosis and autophagy are unclear. According to a previous report, NR4A2 interacts with p53 to inhibit the expression of Bax to regulate apoptosis in lung cancer cells^31^. Here, we found that p53/Bax also participated in NR4A2-regulated cardiomyocyte apoptosis by binding to p53 directly. Our data revealed that p53/Bax is the popular downstream factor of NR4A2.

As a member of orphan nuclear receptor family, NR4A2 expression is difficult to regulate through its ligand. MiRNAs can bind to the 3′UTR of target mRNAs to inhibit the expression of specific genes. MiR-212-3p has been shown to have important roles in regulating cardiac hypertrophy^32^. It was reported that miR-132, one member of the miR-132/212 cluster, is induced by H_2_O_2_ to target NR4A2 to aggravate apoptosis^33^, but whether NR4A2 is the target of miR-212-3p in cardiomyocytes was unknown. In our research, we found that miR-212-3p expression decreased in ischemia, and its target NR4A2 increased, inhibiting the apoptosis of cardiomyocytes. Our data suggested that miRNA regulates NR4A2 expression. Furthermore, we reported a new role of miR-212-3p in cardiomyocyte apoptosis and autophagy.

In conclusion, we elucidated the roles of NR4A2 in apoptosis and autophagy in cardiomyocytes exposed to ischemia, and we identified p53/Bax as its downstream effectors. MiR-212-3p is the upstream regulator of NR4A2 expression in ischemic cardiomyocytes. Our data provide a theoretical foundation for the future development of therapeutics for ischemic heart diseases.

## Materials and methods

### Reagents

Dulbecco's modified Eagle's medium (DMEM) was purchased from Gibco (Grand Island, NY, USA). NR4A2 and negative control siRNA were purchased from Ribbio (Guangzhou, China). 3MA, Bafilomycin A1 and NH_4_Cl were purchased from Sangon Biotech (Shanghai, China). Lipofectamine 2000 was purchased from Invitrogen (Carlsbad, CA). NR4A2 antibody was purchased from Santa Cruz Biotechnology (Santa Cruz, CA, USA). Antibodies for caspase3, PARP, LC3, p53 and Bax were from Cell Signaling Technology (Beverly, MA, USA).

### Animals

C57bl/6 mice (25 g) were purchased from the animal center of Shandong University. The animal care and experimental procedures involved in this study were approved by the Institutional Animal Care and Use Committee of Shandong University and agreed with the American Physiological Society’s “Guiding Principles in the Care and Use of Vertebrate Animals in Research and Training”.

### MI model

Male C57bl/6 mice were divided into the following groups: MI and MI plus interfering lentivirus against NR4A2. Both groups had 6 surviving mice. MI was induced by permanent ligation of the left anterior descending coronary artery for 7 days, and heart function was detected by echocardiography as previously described^18^. At the end of the experiment, the mice were killed, and their hearts were collected for analysis.

### Cell isolation, culture and treatment

NRCMs were obtained from the hearts of Wistar rats born within 3 days, as previously reported^34^. NRCMs were cultured in high glucose DMEM with 10% fetal bovine serum (FBS) in humidified air with 5% CO_2_ at 37 °C. H9c2 cells and HEK293 cells were cultured in DMEM with 10% FBS in humidified air with 5% CO_2_ at 37 °C.

To mimic the ischemic situation, NRCMs were exposed to DMEM deprived of glucose and FBS in a hypoxic incubator (95% N_2_ and 5% CO_2_, 37 °C) for 3 h. H9c2 cells were incubated in medium deprived of FBS for 12 h, as previously described^19^.

### Transfection of siRNA or overexpression plasmid

H9c2 or NRCMs were seeded in 60 mm cell culture dishes and cultured in medium without antibiotics overnight. To knockdown the expression of NR4A2, cells at 40% confluence were transfected with NR4A2 siRNA (20, 40, 80 nM) using Lipofectamine 2000 reagent according to the manufacturer’s instructions. At 36 h after transfection, NRCMs and H9c2 were incubated in ischemic conditions for 12 h, as previously described.

To upregulate the expression of NR4A2 or LC3, pcDNA3.1-NR4A2 (4 μg) or pEGFP-LC3 were transfected into H9c2 cells by use of Lipofectamine 2000 reagent (Invitrogen) for 48 h, followed by ischemic treatment for another 12 h; then, the cells were used for analyses.

### Real-time quantitative PCR

Total RNA was extracted from cardiomyocytes using the TRIzol reagent (Invitrogen). The reverse transcription step involved use of oligo (dT) primers, and then underwent qPCR (Roche, LightCycler 2.0 system, Basel, Switzerland) with the primer pair sequences for NR4A2, forward, 5′-TGAGGGTCTGTGCGCTGTT-3′, and reverse, 5′-ACCTTTGCAGCCCTCACAAG-3′, and for glyceraldehyde 3-phosphate dehydrogenase (GAPDH), forward, 5′-GAGTATGTCGTGGAGTCTA-3′ and reverse, 5′-CTAAGCAGTTGGTGGTG-3′. Relative gene expression was normalized to GAPDH levels. The qPCR reactions involved the use of a QuantiTect SYBR Green PCR kit (Qiagen, Germany) and a LightCycler 2.0 system (Roche). Reactions were carried out in a volume of 25 µl with 12.5 µl of 2× SYBR Green PCR Master Mix. The fold change was calculated by the 2-ΔΔCt method with MxPro 4.00 (Stratagene).

### Western blot analysis

Total protein of H9c2 or NRCMs was collected with RIPA lysis buffer on ice. Equal amounts of protein (30 μg) underwent 12% sodium dodecyl sulfate–polyacrylamide gel electrophoresis and were transferred to a polyvinylidene difluoride membrane. After the membrane was blocked with 5% non-fat milk for 1 h, it was incubated with primary antibodies (1:1000) at 4 °C overnight and incubated with horseradish peroxidase-conjugated secondary antibodies (1:5000) for 1 h at room temperature. The immunoreactive bands were developed with an ECL western blotting system. β-Actin was used as the loading control. The relative levels of proteins were analyzed with ImageJ software (National Institutes of Health, USA).

### Immunofluorescence staining

Treated cells were fixed with 4% paraformaldehyde for 10 min at room temperature and incubated with NR4A2 antibodies (1:100) at 4 °C overnight; then, the cells were washed with phosphate-buffered saline and incubated with secondary antibodies (1:200) for 1 h at 37 °C. Nuclei were stained with 4',6-diamidino-2-phenylindole for 15 min at room temperature. Then, the fluorescence was observed by using a Zeiss LSM700 (Carl Zeiss Canada Ltd). Fluorescence intensities were analyzed with ImageJ software.

### Protein Co-IP

H9c2 cells were transfected with an NR4A2 overexpression plasmid or with a negative control plasmid, followed by ischemia treatment. At 60 h after transfection, total protein was collected from cells by using IP lysis buffer (Beyotime, Shanghai, China). After centrifugation at 4 °C, the supernatant was isolated and incubated with protein A/G agarose beads (Beyotime, Shanghai, China) and green fluorescent protein (GFP) antibody (Santa Cruz Biotechnology, Santa Cruz, USA) at 4 °C overnight. The beads were washed 3 times with IP lysis buffer and then eluted with 4× SDS loading buffer. GFP and p53 were detected by western blot assay with their protein-specific antibodies (Cell Signaling Technology, Danfoss, USA).

### Luciferase assay analysis

For the target assay, we constructed luciferase reporter vectors containing the wild-type NR4A2 3′UTR. Then, luciferase reporter plasmids were co-transfected with control or miR-212-3p mimic into HEK293 cells with Lipofectamine 2000 (Invitrogen). The transfected cells were harvested 48 h after transfection, and the luciferase activity was measured with the Dual-Luciferase Reporter System (Promega)^35^.

### Statistical analysis

Data are from at least three independent experiments and expressed as the mean ± standard error. Statistical analysis was performed with the paired Student's *t*-test and analysis of variance with SPSS version 11.5. Differences were considered statistically significant at *P* < 0.05.
